# Adult Attention Deficit Hyperactivity Disorder and Violence in the Population of England: Does Comorbidity Matter?

**DOI:** 10.1371/journal.pone.0075575

**Published:** 2013-09-24

**Authors:** Rafael A. González, Constantinos Kallis, Jeremy W. Coid

**Affiliations:** Forensic Psychiatry Research Unit, Queen Mary, University of London, Barts and The London School of Medicine and Dentistry, London, United Kingdom; Hangzhou Normal University, China

## Abstract

**Background:**

It is unclear whether the association between Attention Deficit/Hyperactivity Disorder (ADHD) and violence is explained by ADHD symptoms or co-existing psychopathology. We investigated associations of ADHD and its symptom domains of hyperactivity and inattention, among individuals reporting violence in the UK population.

**Methods:**

We report data from the Adult Psychiatric Morbidity Survey (2007), a representative sample of the household population of England. A randomly selected sample of 7,369 completed the Adult Self-Report Scale for ADHD and the self-reported violence module, including repetition, injury, minor violence, victims and location of incidents. All models were weighted to account for non-response and carefully adjusted for demography and clinical predictors of violence: antisocial personality, substance misuse and anxiety disorders.

**Results:**

ADHD was moderately associated with violence after adjustments (OR 1.75, *p* = .01). Hyperactivity, but not inattention was associated with several indicators of violence in the domestic context (OR 1.16, *p* = .03). Mild and moderate ADHD symptoms were significantly associated with violence repetition, but not severe ADHD where the association was explained by co-existing disorders. Stratified analyses further indicated that most violence reports are associated with co-occurring psychopathology.

**Conclusions:**

The direct effect of ADHD on violence is only moderate at the population level, driven by hyperactivity, and involving intimate partners and close persons. Because violence associated with severe ADHD is explained by co-existing psychopathology, interventions should primarily target co-existing disorders.

## Introduction

Attention deficit-hyperactivity disorder (ADHD) is associated with criminality [1], including violent crime [2] and externalizing behaviours [3,4]. ADHD begins in childhood, often persisting into adulthood [5], with cross-national studies indicating average adult prevalence of 3.4% [6]. Although ADHD is often diagnosed as a unitary construct [7], factor analytic studies consistently confirm a two-dimensional structure of inattention and hyperactivity/impulsivity [8,9], each associated with distinct correlates and executive dysfunction. Hyperactivity symptoms tend to decline more with age, in contrast to inattention, and are associated with conduct problems [10], disinhibition and behavioral dysregulation [11], and substance misuse [12]. Hyperactivity also appears more likely to be associated with violence. However, findings differ on the role of comorbid psychopathology in this association. Inattention is associated with low educational achievement [13,14], and cognitive compromise related to working memory, monitoring, and executive difficulties [11], which may impair regulation of interpersonal interactions in certain circumstances, leading to violent altercations.

Nevertheless, a more complex association is suggested by longitudinal studies showing that childhood ADHD is a risk factor for a wide range of adverse psychiatric outcomes in adulthood associated with violence, including addictive, mood and anxiety disorders, and antisocial personality disorder (ASPD) [15,16,17]. Correspondingly, studies which adjust for childhood conduct disorder (CD), a common precursor of ASPD [18,19], or exclude participants with co-occurring CD [20], demonstrate that associations with violence are almost entirely explained by coexisting ASPD. Recent studies conducted in prison, however, provide evidence of associations between hyperactivity and violent breaches of custody [21], as well as violent critical incidents [22] above and beyond CD/ASPD.

Because violence is increasingly recognized as a major public health problem [23] requiring intervention from healthcare professionals, and the potential impact on violence from neurodevelopmental psychopathology has not been systematically studied at the population level [19], we investigated associations between ADHD and violence in the adult household population of England in a representative survey carried out in 2007 [24]. Our aims were to determine the extent to which ADHD contributes to the public health problem of violence and implications for further targeted interventions on ADHD to reduce violence. We investigated: (1) prevalence of self-reported violence associated with ADHD, its severity, victims and location of violent incidents, (2) the independent associations between symptom dimensions of ADHD-inattention and hyperactivity/impulsivity, and (3) whether the direct associations of ADHD with violence were independent of coexisting psychopathology, including ASPD and substance dependence.

## Methods

### Sample and Participants

The Adult Psychiatric Morbidity Survey 2007 (APMS, 2007) recruited adults aged 16 and over living in private households in England. Field work was carried out between October 2006 and December 2007 and detailed information is published elsewhere [24]. A multistage stratified probability sampling design was adopted based on the small user Postcode Address File. One adult aged 16 years or more was selected from each household for interview using the Kish Grid method [25]. Phase one data were collected by lay interviewers. A total of 7,403 adults completed first phase interviews, representing 57% of those eligible and originally approached. There is no information regarding mental health status of non-respondents. However, data were weighted to account for non-response. Sample weights were also assigned to take into account different probabilities in household selection. All models were corrected for area clusters based on postcodes. Out of the 7,403 participants, 7,369 completed the violence self-report questions, representing the sample for this study.

### Ethical Statement

Ethical approval for the Adult Psychiatry Morbidity Survey (APMS 2007) was obtained from the Royal Free Hospital and Medical School Research Ethics Committee (Ethical approval reference number 06/Q0501/71), one of the Research Ethics Committees of the National Research Ethics Service for non-clinical populations.

Consent for participation was obtained in written form by participants. The APMS 2007 was designed to study the English adult population, and individuals aged 16-17 can legally consent as adults for medical and research purposes in the UK (e.g., Mental Capacity Act, 2005).

### Measures

The 6- item version of the Adult Self Report Scale (ASRS) [26] based on DSM-IV diagnostic criteria [7] was included as a measure of ADHD for the first time in a UK population survey. The ASRS is well recommended for community epidemiological research as it has strong concordance with clinical diagnoses, with a reported area under the curve (AUC) of 0.90. Four items are related to inattention and two to hyperactivity. Items are based on a 5-point likert scale [26,27]. We used the ASRS-6 scoring and classification methods recommended of a binary cut-off of 13 or more [28], and four severity strata according to guidelines: 0-9, Stratum I; 10-13, Stratum II; 14-17, Stratum III; 18-24, Stratum IV. To explore relative contributions of ADHD symptoms, the sum of response scores was calculated for inattention items (items 1-4) and hyperactivity items (items 5-6), an adequate method recently employed by [29] on population data.

Common mental disorders were assessed using the Clinical Interview Schedule-Revised (CIS-R) [30]. This measures non-psychotic symptoms in the past week and provides several derived diagnostic categories. For adjustments in subsequent analyses, the Generalised Anxiety Disorder, Panic Disorder, Obsessive-Compulsive Disorder and Mixed Anxiety-Depressive Disorder categories were combined into a single category of Anxiety Disorders.

ASPD was measured using the self-report version of the Structured Clinical Interview for DSM-IV Axis II disorders [31,32]. The algorithm follows strictly the ASPD DSM-IV criteria, including positive screening for CD.

Alcohol dependence was examined using the Severity of Alcohol Dependence Questionnaire (SADQ) [33], which estimates prevalence of alcohol dependence symptoms in the past 6 months. Questions about drug use were asked in a computer-assisted self-completion interview containing lifetime experience of 13 different types of illicit drugs, together with patterns of use over the last year. Five questions derived from the Diagnostic Interview Schedule (DIS) [34], were used to measure substance dependence.

Positive psychotic disorder classification was assessed via the Psychosis Screening Questionnaire (PSQ), which includes 5 main and secondary items covering mania, thought insertion, paranoia and hallucinations in the past year [35].

### Demographic Covariates

Social class was based on the UK Registrar General’s Classification [36] which uses the most recent occupation of the head of household: I - professional, II - managerial, IIIA - skilled non-manual, IIIB - skilled manual, IV – partly skilled, V- unskilled. These were combined in three categories: I & II (upper middle class), III (lower middle and skilled working class) and IV & V (less skilled and unskilled). Additional socio-demographic covariates included gender, age, marital status and ethnicity (white, black/African origin, Indian subcontinent, other).

### Violence

Participants were asked about violent behaviour in the section which assessed ASPD. The first question was: “Have you been in a physical fight or deliberately hit anyone in the past 5 years?” Additional questions then included information on violence severity, including: repetition (5 or more incidents), violent when intoxicated, victim or perpetrator injured, police involvement, and minor violence (when none of the preceding occurred). Additional information included: victims and location of incidents [37].

### Statistical Analyses

Weighted (N) frequencies and proportions were reported on all categorical variables. Group associations were established using binary logistic regression with Odds Ratios (OR) as a measure of magnitude. Multiple categorical predictor covariates were assigned a reference category against which other categories were contrasted.

Two approaches examined the associations of ADHD with dependent binary measures of violence. In the first, the exposure was a binary measure of ADHD, based on a cut-off of 13 or more [28], and by the four severity strata (I – IV) previously described. In the second, considering our expectation that hyperactivity as a potential predictor of violence had a greater effect than inattention, we tested hyperactivity and inattention scores as an alternative to the ADHD categorical classifications using unequally weighted symptom dimension scores.

When testing for number of violent incidents (count variable), the Negative Binomial Regression (NBR) was applied. This model is suited to account for observed and unobserved heterogeneity when using count variables by under fitting the dispersion [38]. ADHD was treated as exposure using the ADHD binary classification, the four severity strata, and the hyperactivity and inattention continuous scores. The Incidence Rate Ratio (IRR) was used as the measure of effect in the NBR models.

Considering the high levels of comorbidity of adult ADHD [15], and to further explore the impact of coexisting disorders on associations with violence, we performed logistic regressions for binary ADHD on violence stratifying by individuals with and without comorbid ASPD, alcohol and drug dependence and anxiety disorders separately.

All statistical analyses were adjusted by including covariates in each model simultaneously. Adjustments included demographic factors, ASPD, drug dependence, alcohol dependence and anxiety disorders. To adjust effects of selecting one individual per household and under-representation of certain subgroups, and to account for any deviation from selecting a simple random sample, all variance estimates were weighted. Details of the procedures used in weighting have previously been described [24]. All models employed robust standard errors to adjust for clustering of individuals within postcodes. All analyses were performed using Stata version 12 (StataCorp.).

## Results

### Demographic Characteristics

Of 7,369 respondents, 614 (8.4%) reported any violence in the past 5 years. Socio-demographic factors of male gender, marital status other than married, and social class lower than I & II were significantly associated with violence risk, whereas any age category above 34 years were protective. All clinical and personality covariates significantly contributed to violence after adjustments, with the exception of alcohol dependence with a weak association (P < 0.10) and screening positive for psychosis (Table 1).

**Table 1:** Demographic and clinical covariates in association with ‘any violence in the past 5 years’ and ADHD classification.

	Any violence in the past 5 years				ADHD classification		
	614 (8.4%)				424 (5.7%)		
Covariate	N (%) reported	Adjusted OR (95% CI)	P Value		N (%) reported	Adjusted OR (95% CI)^a^	P Value
Sex							
Female	173 (4.6)	Reference			205 (5.4)	Reference	
Male	441 (12.3)	3.14 (2.42-4.07)	**<0.001**		219 (6.1)	1.41 (1.09-1.81)	**0.008**
Age							
16-34	448 (19.9)	Reference			189 (8.3)	Reference	
35-54	141 (5.4)	.32 (.24-.42)	**<0.001**		171 (6.5)	1.01 (.74-1.38)	0.94
55 or more	25 (1.0)	.06 (.04-.10)	**<0.001**		64 (2.6)	.47 (.32-.68)	**<0.001**
Marital status							
Married/Cohabit.	235 (4.6)	Reference			225 (4.4)	Reference	
Single	340 (20.4)	1.61 (1.20-2.16)	**0.001**		157 (9.4)	1.10 (.79-1.53)	0.58
Separated	39 (7.2)	1.70 (1.14-2.54)	**0.009**		42 (7.6)	1.16 (.82-1.64)	0.41
Social class							
I & II	114 (4.3)	Reference			110 (4.2)	Reference	
III (M & NM)	264 (9.5)	2.10 (1.55-2.84)	**<0.001**		158 (5.6)	1.27 (.93-1.73)	0.13
IV & V	149 (10.5)	2.15 (1.51-3.08)	**<0.001**		101 (7.1)	1.40 (.99-1.98)	0.06
Ethnicity							
White	550 (8.3)	Reference			366 (5.5)	Reference	
Black	21 (9.3)	.87 (.43-1.76)	0.69		22 (9.9)	1.67 (.89-3.14)	0.11
Indian subcont.	19 (6.9)	.50 (.23-1.08)	0.08		10 (3.7)	.66 (.29-1.50)	0.32
Other	19 (8.7)	.78 (.37-1.63)	0.51		20 (9.4)	1.35 (.66-2.76)	0.41
ASPD	80 (41.1)	2.26 (1.40-3.64)	**0.001**		45 (22.6)	1.72 (1.02-2.91)	**0.04**
Drug dependence	111 (44.2)	3.36 (2.18-5.16)	**<0.001**		46 (18.1)	1.64 (.93-2.91)	0.09
Alcohol depend.	106 (24.5)	1.38 (.95-2.00)	0.09		74 (17.0)	1.70 (1.09-2.65)	**0.02**
Anxiety disorder	165 (15.2)	2.07 (1.54-2.80)	**<0.001**		228 (20.8)	6.89 (5.37-8.85)	**<0.001**
Psychosis	23 (24.5)	1.77 (.83-3.81)	0.14		33 (33.6)	3.13 (1.78-5.53)	**<0.001**

Note: All **weighted** percentages and estimates (N = 7,369)

^a^Adjusted for the following socio-demographic and clinical characteristics: gender, age, marital status, social class and ethnicity; alcohol and drug dependence, anxiety disorders and antisocial personality disorder (ASPD).

Overall prevalence of ADHD in the sample was 5.7%. The older age group (55 or more) showed significantly lower rates of ADHD. Anxiety disorders, ASPD and alcohol dependence were significantly associated with ADHD, whilst drug dependence showed a weaker association (P < 0.10) in the adjusted covariates model for ADHD (all covariates simultaneously).

### Main Associations of ADHD with Violence

Table 2 shows the number of respondents who reported violence when intoxicated with alcohol and/or drugs, involvement in 5 or more incidents, injury to victims, or who were injured themselves. ADHD was initially associated with all violent outcomes. Following adjustments, this effect was only observed for having been involved in any violence in the past 5 years and in violent events considered minor.

**Table 2:** Adjusted associations of ADHD and symptom dimensions on measures of any violence in the past 5 years, severity and repetition, victim types and locations.

outcomes	N (%) violent	ADHD^a^ OR (CI 95%)	P Value	Hyperactivity OR (CI 95%)^b^	P Value	Inattention OR (CI 95%)^b^	P Value
Any violence	614 (8.4)	1.75 (1.14-2.68)	**0.01**	1.15 (1.08-1.23)	**<0.001**	1.06 (1.02-1.11)	**0.003**
Intoxicated	263 (3.6)	1.34 (0.72-2.48)	0.35	1.15 (1.04-1.26)	**0.004**	1.03 (0.97-1.10)	0.30
Minor violence	247 (3.3)	2.54 (1.48-4.34)	**0.001**	1.18 (1.07-1.30)	**0.001**	1.11 (1.05-1.18)	**0.001**
Severity							
5 > incidents	98 (1.3)	1.42 (0.49-4.15)	0.52	1.16 (0.96-1.40)	0.13	1.01 (0.92-1.11)	0.83
Victim injured	172 (2.3)	0.88 (0.37-2.14)	0.78	1.10 (0.98-1.24)	0.11	0.99 (0.92-1.06)	0.81
Perp. injured	204 (2.8)	1.10 (0.55-2.20)	0.80	1.06 (0.95-1.18)	0.30	1.00 (0.94-1.07)	0.96
Police involved	177 (2.4)	1.19 (0.56-2.55)	0.65	1.15 (1.02-1.28)	**0.02**	0.97 (0.90-1.04)	0.39
Victim types							
IPV	115 (1.6)	1.52 (0.70-3.28)	0.29	1.16 (1.01-1.32)	**0.03**	1.05 (0.97-1.14)	0.20
Family	91 (1.2)	1.77 (0.70-4.44)	0.23	1.26 (1.08-1.46)	**0.003**	1.04 (0.94-1.16)	0.40
Friend	132 (1.8)	1.53 (0.60-3.89)	0.37	1.18 (1.02-1.37)	**0.03**	1.06 (0.97-1.16)	0.19
Person known	195 (2.6)	1.16 (0.57-2.36)	0.69	1.10 (0.98-1.23)	0.12	1.03 (0.95-1.11)	0.52
Stranger	300 (4.1)	1.15 (0.64-2.08)	0.63	1.07 (0.97-1.18)	0.15	1.04 (0.99-1.10)	0.15
Locations							
Own home	123 (1.7)	1.75 (0.87-3.52)	0.12	1.21 (1.06-1.39)	**0.004**	1.02 (0.94-1.11)	0.63
Else’s home	61 (0.8)	1.25 (0.49-3.17)	0.64	1.21 (1.05-1.40)	**0.01**	0.93 (0.83-1.03)	0.17
Bar/pub	183 (2.5)	1.50 (0.74-3.05)	0.26	1.17 (1.05-1.31)	**0.006**	1.01 (0.93-1.10)	0.80
Workplace	21 (0.3)	2.79 (0.83-9.39)	0.10	1.25 (1.01-1.54)	**0.04**	1.14 (0.98-1.32)	0.09

Note: All **weighted** percentages and estimates (N = 7,369)

^a^Adjusted for the following socio-demographic and clinical characteristics: gender, age, marital status, social class and ethnicity; alcohol and drug dependence, anxiety disorders and antisocial personality disorder (ASPD).

^b^Adjusted model with nominal ADHD replaced by continuous hyperactivity and inattention scores, entered simultaneously, as exposure variables.

When using four ADHD symptom strata with none or few symptoms (stratum I) as reference, strata II, III and IV all were significantly associated with any violence (OR 2.44, 4.75 and 5.04 respectively, all P <0.001). Nevertheless, after fully adjusting for demography and psychopathology only those in stratum III had significant associations with violence (OR 1.88, P = 0.01).

There were no significant associations between ADHD and either victims or locations of violent incidents following adjustments (Table 2).

### ADHD Symptom Dimensions and Violence: Hyperactivity and Inattention

To test independent contributions of hyperactivity and inattention, we developed adjusted models, replacing the ADHD binary variable with continuous scores of hyperactivity and inattention. Considering the correlation between the inattention and hyperactivity scales was 0.36 (P < 0.001), they were entered in models simultaneously (i.e., adjusted for each other). Table 2 also shows that aside from ‘any violence’, inattention was associated only with minor violence. However, there were additional independent associations with hyperactivity, including any violence, violent when intoxicated, and police involvement. Direct associations with hyperactivity were also observed for specific victim types, including those in intimate partner violence (IPV), family members, and friends. Violence was more likely to occur in the participant’s or another person’s home, in a bar/pub or workplace.

### ADHD and Number of Violent Incidents

**Figure 1:**
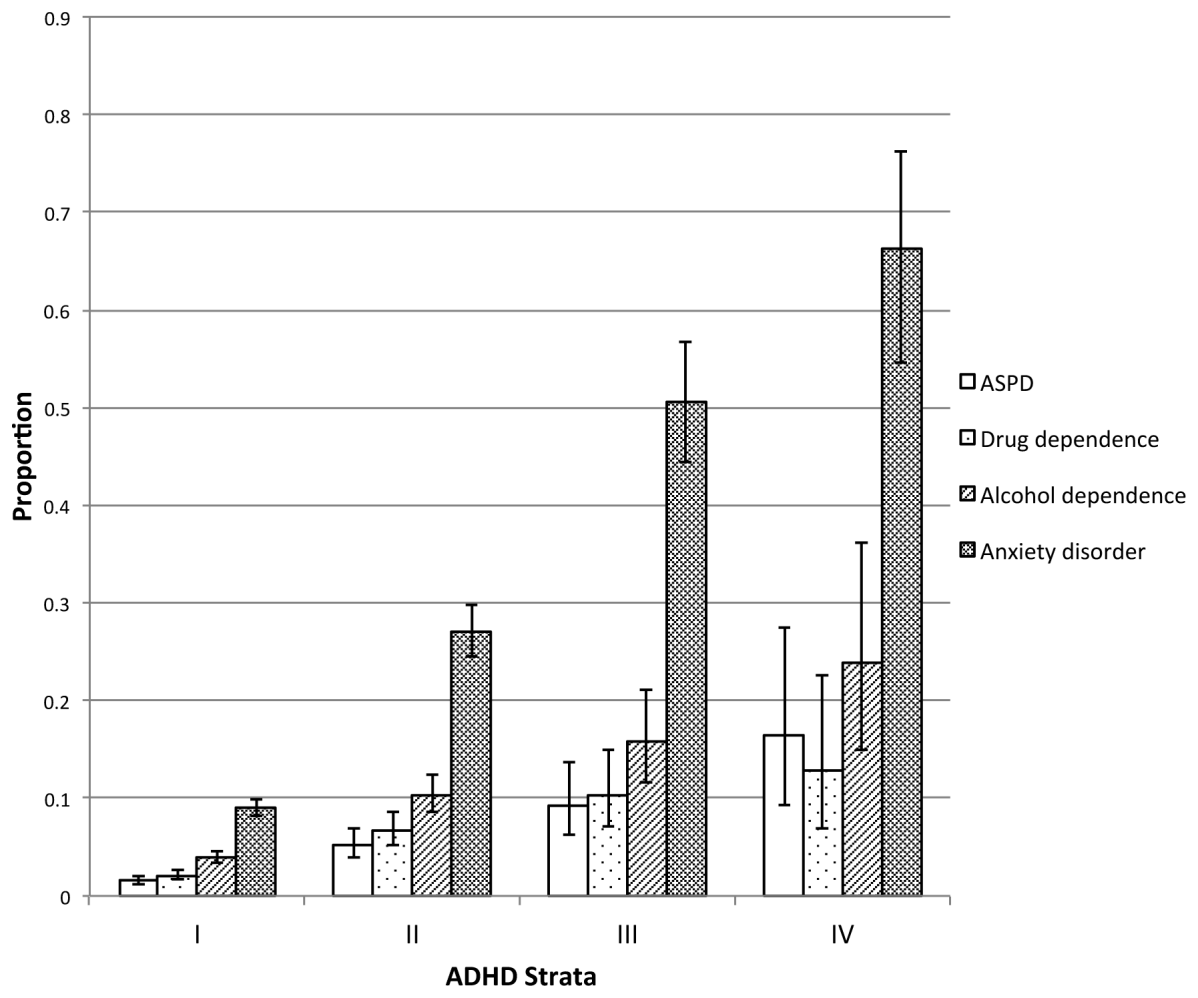
Proportion of psychiatric morbidity by ADHD severity. Shown are the gradient for the prevalence of psychiatric morbidity by increasing levels of ADHD severity (strata I-IV). Design-based tests of linear trend for co-occurring clinical syndromes are the following: ASPD F = 113.8, Drug Dependence F = 99.2, Alcohol Dependence F = 128.1 and Anxiety disorders F = 466.8 (all P < 0.001).

To explore the effect of ADHD on number of violent incidents, we used negative binomial regression (NBR) models. Table 3 shows that the binary measure of ADHD was not significantly associated following adjustments. However, using ADHD strata I-IV (stratum I as reference), an increasing linear effect of ADHD was observed in each category for the unadjusted model. Following adjustment, however, stratum II and III yielded significant incidence rate ratios (IRR) on violent incidents, but this effect was attenuated for stratum IV. We strongly suspected this attenuation was due to higher comorbidity rates among those in ADHD stratum 4, therefore, we tested whether there was a significant progression of levels of psychiatric morbidity with increasing ADHD severity. All categories of psychiatric disorders showed a significant linear trend (P <0.001) by ADHD strata. Figure 1 shows the proportion of comorbid psychopathology within each ADHD stratum. For instance, the percentage of cases with ASPD increased from 9% in stratum III to 16% in stratum IV, whilst anxiety disorders increased from 51% to 66%.

**Table 3:** Associations of ADHD symptoms and severity with number of violent incidents: Incidence Rate Ratios (IRR)^c^.

Exposure	N (%)	Unadjusted IRR (CI 95%)	P Value	Adjusted^a^ IRR (CI 95%)	P Value
ADHD					
No	6965 (94.3)	Reference		Reference	
Yes	424 (5.7)	3.37 (2.34-4.86)	**<0.001**	1.24 (.83-1.86)	.29
ADHD strata (I-IV)					
I	5627 (76.2)	Reference		Reference	
II	1338 (18.1)	2.80 (1.94-4.03)	**<0.001**	1.57 (1.06-2.33)	**.02**
III	339 (4.6)	4.16 (2.83-6.10)	**<0.001**	1.63 (1.06-2.51)	**.03**
IV	86 (1.2)	6.00 (2.58-13.95)	**<0.001**	.82 (.46-1.46)	.49
Hyperactivity and Inattention model^b^					
H score	-	1.30 (1.21-1.40)	**<0.001**	1.17 (1.07-1.28)	**<0.001**
I score	-	1.13 (1.07-1.19)	**<0.001**	1.01 (.97-1.06)	0.60

Note: All **weighted** percentages and estimates (N = 7,369)

^a^Adjusted for the following socio-demographic and clinical characteristics: gender, age, marital status, social class and ethnicity; alcohol and drug dependence, anxiety disorders and antisocial personality disorder (ASPD).

^b^Model with ADHD replaced by continuous hyperactivity and inattention scores, entered simultaneously, as exposure variables.

^c^Negative Binomial Regression models (NBR)

The association between ADHD symptom dimensions and number of violent incidents were also investigated. The adjusted NBR model for continuous scores of hyperactivity and inattention demonstrated a significant association with hyperactivity but not inattention (Table 3).

### Impact of Co-Existing Disorders on the Association of ADHD with Any Violence

**Figure 2:**
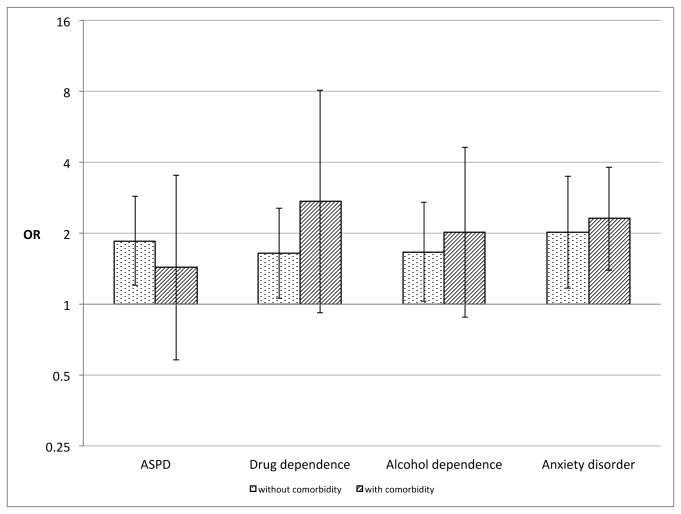
Impact of psychiatric comorbidities on the association of ADHD with any violence in the past 5 years. Error bars that cross vertical gridline 1 indicate no significant association (OR 95% CI).

We performed logistic regression analyses stratified by comorbidities (presence/absence) in order to examine the impact of specific co-occurring disorders on the association of ADHD with reports of any violence. Figure 2 shows the results of these adjusted analyses. ADHD was significantly associated with reports of any violence in absence of ASPD (OR 1.86, P < 0.01), Drug dependence (OR 1.65, P < 0.05), Alcohol dependence (OR 1.67, P < 0.05) and anxiety disorders (OR 2.02, P < 0.05). However, with the exception of anxiety disorders, which marginally increased the magnitude of the association of ADHD with violence, the rest of co-occurring disorders attenuated the associations with violence.

## Discussion

We found that categorically defined ADHD was only moderately associated with violence at the population level following adjustments for demographic factors and coexisting psychopathology. Furthermore, the association was only for minor violent incidents where no one was injured and the police were not involved. After separating ADHD into two continuous measures based on symptom dimensions, inattention demonstrated little or no impact on violence in the population and involved only minor incidents. However, hyperactivity was independently associated with violent incidents resulting in police involvement, with reports of victims in close relationships, taking place in the perpetrator’s or someone else’s home, as well as violence in pubs and bars. Hyperactivity, but not inattention, was additionally associated with repetition of violent incidents.

Finally, we found a more complex relationship between increasing levels of severity of ADHD with any and repetitive violence. Unadjusted models demonstrated a linear relationship with severity of ADHD. Following adjustments, moderate levels of severity still demonstrated associations with violence, but at the most severe level (stratum IV) there were no direct associations. Further examination of our data suggests that the association with violence and its repetition among participants in stratum IV was entirely explained by coexisting conditions of ASPD, alcohol and drug dependence, and anxiety disorders, which were highly prevalent among participants with severe ADHD. We thereby conclude that repetitive violence among persons with severe ADHD is associated with multiple forms of coexisting psychopathology but not ADHD.

### Explaining Associations between ADHD and Violence

Our findings are consistent with reports that the relationship between hyperactivity and serious violence (measured by criminal convictions) is mediated by ASPD [20] and other coexisting psychopathology [19]. We have previously demonstrated that ASPD and substance dependence are independently associated with more severe and repetitive forms of violence at the population level, violence involving multiple victim types, and in different locations [37]. Our models in this study were driven primarily by the influence of coexisting disorders, as well as demographic influences of being male and of lower social class.

Symptom onset of ADHD usually precedes CD and substance misuse, increases risk of developing these conditions, and frequently amplifies their severity [39,40]. However, due to the cross-sectional nature of our data, it was not possible to explore potential mediation effects of these disorders on ADHD and violence. Nevertheless, evidence suggests that mediation effects exist [17,41] and include the relationship between ADHD and adverse psychosocial outcomes [3,42], and sociodemographic variables such as younger age and male sex, also linked to increased risk of violence [43]. Similarly, the heterogeneous symptom presentation of ADHD, combining behavioural with emotional disturbance, may render individuals more vulnerable to additional mental disorders, with the possibility of specific pathways to violence [44]. Future investigation may show whether ADHD indirectly influences violent outcomes by deterring the development of behavioural and emotional regulatory traits that would otherwise reduce the risk of violence. Recent findings indicate that low level or absence of ADHD symptomatology has a protective effect against aggression and violence [45].

### Hyperactivity, Inattention, and Violence towards Close Persons

Our findings suggested that direct associations of ADHD are restricted to violence towards persons in intimate relationships, family members, and friends. A representative US longitudinal study demonstrated that ADHD is consistently associated with violence in intimate relationships [46]. After controlling for CD, associations of hyperactivity and inattention with IPV remained. However, inattention was only related to violence without injury, (corresponding to minor violence in our study) whereas hyperactivity/impulsivity was specifically associated with intimate partner violence resulting in injury [46]. Although not related to injury in our study, hyperactivity was associated with repetitive violent incidents which increased risk of serious violence.

We did not measure motivating factors or other qualitative characteristics such as whether violence was reactive or defensive to aggression initially directed towards the participant, or whether proactive, predatory, or instrumental. The latter characteristics are more common among individuals with ASPD [47,48], result in more serious injuries [49], thereby resulting in criminal convictions, and involve strangers as well as family members [37]. Further investigation may indicate whether proactive and instrumental characteristics of violence are unrelated to ADHD, whereas risk of reactive violence is increased towards close persons, specifically in association with hyperactivity. Impairment of cognitive functioning through inattention with added risks from intoxication could also result in minor violent incidents in reaction to minor stress and perceived provocation.

Our findings also confirm the magnitude of the association of the hyperactivity construct with behavioural disturbance and impulsivity. Hyperactivity was recently linked to increased risk of aggression amongst adults [50]. These authors argued that hyperactivity, even in absence of CD, represents the marker in the association of ADHD with violence. According to this view, aggression and violence stem from deficient self-regulatory processes, in a similar fashion to the difficulties associated with ADHD, including response disinhibition, failure to delay gratification, and emotional reactivity [51] and dysregulation [52,53].

### Limitations

The findings in this report should be interpreted in the context of several methodological limitations. Psychiatric diagnostic classification was derived from self-report scales and not by structured clinical diagnostic interviews. Interpretations regarding ADHD and co-occurring disorders should consider possible false positives in all estimates. Violence and psychiatric morbidity were not contrasted or corroborated against other sources of information, such as official offence records or collateral reports. Self-report in this aspect might have underestimated the prevalence for violence, partly due to social desirability. There is no information regarding childhood diagnosis of ADHD.

Only 57% of all originally approached participated in the survey. It is likely that these differed from non-responders in a way that is not possible to evaluate. However our estimates were weighted to account for non-response based on differences from census data.

Another limitation is related to temporal relation of our predictor and outcome assessments. Violence was assessed over a period over 5 years, and whilst ADHD assumes a stable trajectory of symptoms from childhood, certain diagnostic covariates were assessed over a shorter time span (e.g., last 12 months). Additionally, the cross-sectional design does not allow conclusions on causal associations between ADHD and violence, or the investigation of potential mediating effects of ASPD and/or substance misuse on these associations.

High levels of comorbidity amongst individuals with ADHD, overlap of symptoms across co-occurring disorders, and overall heterogeneity of symptom presentation indicated limitations in assessing categorical adult ADHD, with potential confounding from dimensions of ADHD on the different associations with violence. Our findings indicate that ADHD should be investigated further from a dimensional perspective to gain better understanding into its underlying associations with co-occurring disorders, longitudinal development, and to inform targeted interventions [54,55].

### Interventions for ADHD and Violence

ADHD is an impairing [56] lifelong condition in adults and is under-diagnosed and untreated in many European countries [57]. Violence towards others is primarily related to coexisting psychopathology. However, because comorbidity is highly prevalent among those with ADHD, violence should be seen as prominent among problems resulting in burden of care and high costs [58,59]. Symptoms of ADHD can be treated effectively with medication in both children and adults [60,61,62,63]. However, doubts were cast on the effectiveness of medication for violence by a large randomised clinical trial among children. By 36 months, more days of prescribed medication were unexpectedly associated with more serious delinquency [64]. In contrast, the Swedish case registers study of adults suggested that rates of criminality were lower whilst receiving medication, and lowest for crimes of violence [65]. However, our findings put into question whether these effects on violence among adults were due to medication. Because medication was restricted to the most severe cases of ADHD in Sweden, it is likely that their violent offending was due to coexisting psychopathology and not ADHD. Our findings suggest firstly, that because the direct associations with ADHD are minor, and therefore unlikely to lead to violent incidents resulting in criminal convictions, and secondly, are more likely to involve close persons who are less likely to report violence, medication was unlikely to explain these effects.

Nevertheless, our findings have two important implications for targeted interventions for persons with ADHD to reduce violence. Firstly, medication treatment may be important if the aim is to reduce minor violence experienced by close persons, including intimate partners, family, and friends. The burden of care resulting from even minor violence directed towards close persons should not be underestimated. Secondly, further trials of medication on violent behaviour should be extended to those with low and moderate levels of ADHD where we have shown that symptoms of hyperactivity have an independent effect. Lastly, psychological interventions with focus on the development of executive, regulatory techniques and coping skills may play a critical role in managing impulsive behaviour associated with ADHD and its common co-occurring disorders [66,67].
